# Epidemiology characteristics of human coronaviruses in patients with respiratory infection symptoms and phylogenetic analysis of HCoV-OC43 during 2010-2015 in Guangzhou

**DOI:** 10.1371/journal.pone.0191789

**Published:** 2018-01-29

**Authors:** Su-fen Zhang, Jiu-ling Tuo, Xu-bin Huang, Xun Zhu, Ding-mei Zhang, Kai Zhou, Lei Yuan, Hong-jiao Luo, Bo-jian Zheng, Kwok-yung Yuen, Meng-feng Li, Kai-yuan Cao, Lin Xu

**Affiliations:** 1 Department of Microbiology, Zhongshan School of Medicine, Sun Yat-sen University, Guangzhou, Guangdong Province, China; 2 Key Laboratory of Tropical Disease Control, Ministry of Education, Sun Yat-Sen University, Guangzhou, Guangdong Province, China; 3 Clinical Laboratory and Institute of Medical Genetics, Women and Children's Healthcare Hospital of Zhuhai City, Zhuhai, Guangdong Province, China; 4 Sun Yat-sen University—University of Hong Kong Joint Laboratory of Infectious Disease Surveillance, Sun Yat-sen University, Guangzhou, Guangdong Province, China; 5 Medical ICU, the First Affiliated Hospital, Sun Yat-sen University, Guangzhou, Guangdong Province, China; 6 Department of Microbiology, University of Hong Kong, Hong Kong SAR, China; Deutsches Primatenzentrum GmbH - Leibniz-Institut fur Primatenforschung, GERMANY

## Abstract

Human coronavirus (HCoV) is one of the most common causes of respiratory tract infection throughout the world. To investigate the epidemiological and genetic variation of HCoV in Guangzhou, south China, we collected totally 13048 throat and nasal swab specimens from adults and children with fever and acute upper respiratory infection symptoms in Gunazhou, south China between July 2010 and June 2015, and the epidemiological features of HCoV and its species were studied. Specimens were screened for HCoV by real-time RT-PCR, and 7 other common respiratory viruses were tested simultaneously by PCR or real-time PCR. HCoV was detected in 294 cases (2.25%) of the 13048 samples, with most of them inpatients (251 cases, 85.4% of HCoV positive cases) and young children not in nursery (53.06%, 156 out of 294 HCoV positive cases). Four HCoVs, as OC43, 229E, NL63 and HKU1 were detected prevalent during 2010–2015 in Guangzhou, and among the HCoV positive cases, 60.20% were OC43, 16.67% were 229E, 14.97% were NL63 and 7.82% were HKU1. The month distribution showed that totally HCoV was prevalent in winter, but differences existed in different species. The 5 year distribution of HCoV showed a peak-valley distribution trend, with the detection rate higher in 2011 and 2013 whereas lower in 2010, 2012 and 2014. The age distribution revealed that children (especially those <3 years old) and old people (>50 years) were both high risk groups to be infected by HCoV. Of the 294 HCoV positive patients, 34.69% (101 cases) were co-infected by other common respiratory viruses, and influenza virus was the most common co-infecting virus (30/101, 29.70%). Fifteen HCoV-OC43 positive samples of 2013–2014 were selected for S gene sequencing and phylogenetic analysis, and the results showed that the 15 strains could be divided into 2 clusters in the phylogenetic tree, 12 strains of which formed a separate cluster that was closer to genotype G found in Malaysia. It was revealed for the first time that genotype B and genotype G of HCoV-OC43 co-circulated and the newly defined genotype G was epidemic as a dominant genotype during 2013–2014 in Guanzhou, south China.

## Introduction

Coronaviruses, a genus of the *Coronaviridae*, are enveloped single positive-stranded RNA viruses, which have the largest viral genome (26-33kb) among the RNA viruses [1–2]. The *Coronaviridae* family comprises two subfamilies as *Coronavirinae* and *Torovirinae*, and *Coronavirinae* can be subdivided into four groups, the alpha, beta, gamma and delta coronaviruses by phylogenetic clustering [1–4]. Coronaviruses have been identified to infect mammals and birds including bat, mouse, alpacas, swine, dog, cattle, chicken, horse, and also human, etc. [1–4], and can cause a variety of diseases including gastroenteritis and respiratory tract infection, etc. [4] In humans, coronaviruses (HCoV) are proved to cause respiratory tract infection, most frequently common cold, but can also cause severe respiratory illness including severe acute respiratory syndrome (SARS) and Middle East respiratory syndrome (MERS) [4–7]. By now, six human coronavirus species have been identified, including OC43, 229E, NL63, HKU1, SARS-CoV, and MERS-CoV [4–9]. HCoV-OC43 and HCoV-229E were identified nearly 50 years ago, which mainly cause common cold in humans, and the recently identified NL63 and HKU1 are reported to cause mild respiratory tract infection, and these 4 coronaviruses can also cause severe lower respiratory tract infections in young children or elderly adults with underlying diseases [8–9]. HCoV-NL63 is also associated with acute laryngotracheitis (croup) [8–9]. SARS-CoV, a group 2b β-coronavirus, initially emerged in 2002–2003 in Guangdong province, south China, which caused severe lower respiratory tract infection with high morbidity and mortality (approaching 50% in individuals over 60 years of age) known as SARS [10–11]. In 2012, a novel group 2c β-coronavirus coronavirus MERS-CoV was firstly identified in Saudi Arabia [12–13]. It is the causative agent in a series of highly pathogenic lower respiratory tract infections with high mortality (20% to 40%), which is mainly epidemic in the Middle East, but also brought an outbreak in South Korea in 2014 [7–8, 12–13].

Coronaviruses could cause both human and veterinary outbreaks owing to their ability to recombine, mutate, and infect multiple species and cell types, so they have the propensity to jump between species. But by now, there is no anti-viral therapeutics that specifically target human coronaviruses, and only limited options are available to prevent coronavirus infections [8–13]. Therefore, surveillance of the epidemiology of human coronavirus (HCoV) and understanding HCoV epidemiological characteristics are very important for the prediction, prevention and control of HCoV infection. Guangdong is the place that SARS-CoV first emerged. But to date, there is little report about the epidemiological characteristics of HCoV and its species in Guangdong, south China. Our previous studies of 7 respiratory viruses showed that the total viral detection rate of HCoV in south China was 2.47% in 2009–2012 [14], but further clarification of the epidemic features of different HCoV species was still needed, and as an important human respiratory virus, the variation features of HCoV-OC43 in Guangzhou at molecular level has not been well addressed.

In the current study, we collected 13048 throat and nasal swab specimens from adults and children with fever and upper respiratory infection symptoms in Guangzhou, south China between July 2010 and June 2015, and the epidemiological features of HCoV and its species were studied, and we also analyzed the phylogenetic feature of HCoV-OC43.

## Materials and methods

### Ethics statement

The research involving human participants was approved by the Medical Ethics Committee of Zhongshan School of Medicine, Sun Yat-sen University, in accordance with the guidelines for the protection of human subjects. Written informed consent was obtained from each participant or the guardian.

### Patients and specimens

Between July 2010 and June 2015, 13048 throat and nasal swabs were obtained from 8602 children (≤15 years old) and 4446 adult patients (>15 years old) who had been admitted to 14 hospitals in Guangzhou, south China. Among the patients, 38.45% (5017) were infants and toddlers younger than 3 years of age (0–35 months). Specimens were only taken from individuals with ≤ 3 days of fever (temperature ≥37.5°C), and with cough, sputum, throat sore, dyspnea and/or other acute respiratory tract infection symptoms. There were 7974 male (61.11%) and 5074 female (38.89%) patients. Male to female ratio of the patients was 1.57:1. Inpatient cases were 8518, and outpatient/ emergency cases were 4530 (see Table 1). Hospitalized to emergency ratio was 1.88:1. Demographic, epidemiology and clinical information including case history, symptoms, physical signs and clinical examination results were collected using a standardized questionnaire. All specimens were added to 2ml VTM (consists of Earle's Balanced Salt Solution (BioSource International, USA), 4.4% bicarbonate, 5% bovine serum albumin, 30 μg/mL amikacin, 100 μg/mL vancomycin, and 40 U/mL nystatin) according to a standard protocol and transported within 8 hr at 4°C to biosafety laboratories of Sun Yat-Sen university, where they were divided into aliquots, and stored at -80°C until further detection.

**Table 1:** Surveillance results of 8 respiratory viruses in Guanzhou, south China during 2010–2015.

Hospital group	Case number	Positive numbers (detection rate %)							
		Flu	PIV	RSV	HMPV	HCoV	ADV	HBoV	HRV
**Outpatient**	**4530**	**1099(25.26)**	**191(4.39)**	**149(3.43)**	**81(1.86)**	**43(0.95)**	**243(5.59)**	**34(0.78)**	**293(6.47)**
Infants and toddlers	671	68(10.13)	57(8.49)	74(11.03)	17(2.53)	4(0.60)	42(6.26)	16(2.38)	53(7.90)
Children	1493	212(14.20)	88(5.89)	51(3.42)	45(3.01)	19(1.27)	140(9.38)	8(0.54)	74(4.96)
Adults	2366	819(34.62)	46(1.94)	24(1.01)	19(0.80)	20(0.85)	61(2.58)	10(0.42)	166(7.02)
**Inpatient**	**8518**	**854(10.02)**	**409(4.80)**	**1276(14.98)**	**234(2.74)**	**251(2.95)**	**460(5.40)**	**155(1.81)**	**508(5.96)**
Infants and toddlers	4346	369(8.49)	283(6.51)	1085(24.97)	145(3.34)	137(3.15)	237(5.45)	129(2.97)	317(7.29)
Children	2092	306(14.63)	73(3.49)	140(6.69)	63(3.01)	63(3.01)	180(8.60)	14(0.67)	94(4.49)
Adults	2080	179(8.61)	53(2.55)	51(2.45)	26(1.25)	51(2.45)	43(2.07)	12(0.58)	97(4.66)
**Total**	**13048**	**1953(14.97)**	**600(4.60)**	**1425(10.92)**	**315(2.41)**	**294(2.25)**	**703(5.39)**	**189(1.45)**	**801(6.14)**
Infants and toddlers/ Children	8602	955(11.10)	501(5.82)	1350(15.69)	270(3.14)	223(2.59)	599(6.96)	167(1.94)	538(6.25)
Adults	4446	998(22.45)	99(2.23)	75(1.69)	45(1.01)	71(1.60)	104(2.34)	22(0.49)	263(5.92)

Infants and toddlers: age ≤3 years; Children: age 3–15 years; Adults: age >15 years. Flu: influenza virus; RSV: respiratory syncytial virus; PIV: parainfluenza virus; ADV: adenovirus; hMPV: human metapneumovirus; HRV: human rhinovirus; HBoV: human bocavirus

All the specimens were tested for human coronaviruses (HCoV), and 12 other common respiratory viruses, including influenza virus types A and B (Flu-A and Flu-B), parainfluenza 1, 2, 3 and 4 (PIV-1, 2, 3 and 4), respiratory syncytial virus A and B (RSV-A and RSV-B), human metapneumovirus (HMPV), adenoviruses (AdV), human rhinovirus (HRV) and human bocavirus (HBoV). All these viruses were tested by real-time PCR, PCR, or RT-PCR methods, as described below. Information of patients whose throat swabs were found positive for HCoV was further analyzed.

### Nucleic acid extraction and reverse transcription

Virus DNA and RNA were extracted from 200 μL of throat and nasal swab specimens using QIAamp MiniElute Virus Spin (QIAGEN, Germany) following the manufacturer’s instructions. Reverse transcription of virus RNA was performed using Thermo Scientific Revert Aid First strand cDNA Synthesis Kit (Thermo, USA), and the procedure was: 25°C 5min, 42°C 60min, followed by 70°C 5min. The cDNA was used for virus detection immediately or stored at -20°C until further use.

### Respiratory viruses screening

Respiratory viruses including Flu-A and -B, PIV -1, -2, -3 and -4, RSV-A and -B, HMPV, AdV, HRV and HBoV were detected by a standard reverse transcription-PCR (RT-PCR), PCR, or real-time PCR methods as described previously [15–25], using specific primers and probes listed in S1 Table.

Screening of HCoV and the 6 species (OC43, 229E, NL63, HKU1, SARS and MERS) used real-time PCR. TaqMan real-time PCR primers and probes (synthesized by Invitrogen, Life Technology, Shanghai) were designed to bind the highly conserved region of HCoV and according species, and analyzed by Primer Express software (Version 3.0, Applied Biosystems, USA) (for primer and probe sequences, see Table 2). Each reaction mixture consisted of 10 μL 2 × iQ Supermix reaction mixture (Bio-Rad), 2 μL of viral cDNA, 0.5 μM each of the forward and reverse primers, and 0.3 μM of the probe, and nuclease-free water to a final volume of 20 μL. For total HCoV, real-time PCR was conducted for 95°C for 5min, followed by 45 cycles of 95°C for 15s, 60°C for 1min. For species 229E/ OC43/NL63/HKU1, real-time PCR was conducted for 95°C for 5min, followed by 45 cycles of 95°C for 15s, 55°C for 1min. For SARS-CoV, real-time PCR was conducted for 50°C 2min, 95°C for 10min, followed by 45 cycles of 95°C for 15s, 60°C for 1min. For MERS-CoV, real-time PCR procedure was 95°C for 5min, followed by 45 cycles of 95°C for 30s, 55°C 15s, 60°C for 45s. All the real-time PCR reactions were performed on an ABI 7500 Real-time PCR system (Applied Biosystems, USA).

**Table 2:** Real-time PCR primers and probes for HCoV and species screening.

Primer/probe	Sequence (5’-3’)	Target Gene	PCR Product (bp)
HCoV-F1	GGTGGYTGGGAYGATATGTTACG	Replicase	100bp
HCoV-F2	GCTRAGCATGATTTCTTTACTTGG		
HCoV-R1	ATGTTGACAAYCCTGTWCTTATGGGTTGGG		
HCoV-R2	CAGARTCATTTATGGTAATGTTAGTAGACA		
HCoV-probe1	FAM-KRTTTGGCATAGCACGATCACA-BHQ		
HCoV-probe2	FAM-CARTYTTKTTCATCAAAGTTACGCA-BHQ		
CoVOC43-F	CGATGAGGCTATTCCGACTAGGT	NP	76bp
CoVOC43-R	CCTTCCTGAGCCTTCAATATAGTAACC		
CoVOC43-probe	FAM-TCCGCCTGGCACGGTACTCCCT-TAMAR		
CoV229E-F	CAGTCAAATGGGCTGATGCA	NP	76bp
CoV229E-R	AAAGGGCTATAAAGAGAATAAGGTATTCT		
CoV229E-probe	VIC-CCCTGACGACCACGTTGTGGTTCA- BHQ		
CoVNL63-F	ACCTAATAAGCCTCTTTCTCAACCC	NP	110bp
CoVNL63-R	GACCAAAGCACTGAATAACATTTTCC		
CoVNL63-probe	CY5-AACACGCTTCCAACGAGGTTTCTTCAACTGAG- BHQ		
CoVHKU1-F	CCTTGCGAATGAATGTGCT	ORF1b	95bp
CoVHKU1-R	TTGCATCACCACTGCTAGTACCAC		
CoVHKU1-probe	CY5-TGTGTGGCGGTTGCTATTATGTTAAGCCTG- BHQ		
CoVSARS-F	CAGAACGCTGTAGCTTCAAAAATCT	ORF1b	67bp
CoVSARS-R	TCAGAACCCTGTGATGAATCAACAG		
CoVSARS- probe	FAM-TCTGCGTAGGCAATCC- BHQ		
MERS-COV-F	ACTGTTGCAGGCGTGTCCATACTTAGCF	ORF1b	108bp
MERS-COV-R	TAGTACCAATGACGCAAGTCGCTCC		
MERS-COV-probe	FAM-CTAATCGCCAGTACCATCAG- BHQ		

### Amplification and sequencing of HCoV-OC43 S gene

For S gene sequencing and phylogenetic analysis of HCoV-OC43, 3 pairs of primers were designed by the Primer Premier 5.0 software to bind relatively conserved regions of S gene (shown in Table 3). The reference sequences used to design the primers included 15 representative strains from different regions of the world and years available in GenBank database. Their GenBank accession numbers are: NC_005147.1, AY585229.1, AY585228.1, KF530099.1, KF530098.1, KF530097.1, KF530096.1, KF530095.1, KF530093.1, KF530091.1, KP198611.1, KJ958218.1, JN129835.1, L14643.1 and Z21849.1. Primers were synthesized by Invitrogen Co., Shanghai, China. The PCR was carried out in a prepared 20 μL reaction mix consisted of 10 μL 2 × Premix Ex Taq (Takara, Dalian, China), 2 μL of template cDNA, 0.5 μM each of the forward and reverse primers. The PCR procedure was: 95°C for 5min, followed by 35 cycles of 95°C for 30s, 50°C to 55°C (see Table 1 for melting temperature of different primers) for 1min, and 72°C for 1min, and a final extension at 72°C for 10min. PCR products for sequencing was purified by agarose gel DNA purification kit (Takara, Dalian, China), and cloned into PMD19-T vector (Takara, Dalian, China). All PCR products used for cloning and sequencing were from three independent PCR reactions. Sequencing was performed by Invitrogen Co., Shanghai, China.

**Table 3:** PCR primers for HCoV-OC43 S gene amplification and sequencing.

Primers	Sequence (5’-3’)	Position^a^	Melting temperature (°C)	PCR Product (bp)
OC43 S-F1	CCCAATGGCAGGAAGGTTGA	24832–25691	50	869
OC43 S-R1	AGCAATGCTGGTTCGGAAGA			
OC43 S-F2	TCTGCGGCCTTTCATGCTAA	25689–26426	55	738
OC43 S-R2	AGCCTCAACGAAACCGACAT			
OC43 S-F3	TGTCGGTTTCGTTGAGGCTT	26411–27327	52	917
OC43 S-R3	TCAGCATTACATACGGCGCT			

^a^ According to GenBank accession number KF530099.1.

### Phylogenetic analysis for HCoV-OC43

The amplified S gene sequences of 15 strains of HCoV-OC43 from 2013–2014 in Guangzhou were comparatively analyzed with reference sequences of 33 representative HCoV-OC43 strains in the GenBank database (including HCoV-OC43 reference sequence NC_005147, and sequences from different countries and different years). These sequences were aligned by the Clustal X program, and a phylogenetic tree was constructed using the MEGA 5.0 software by neighbor-joining method using Kimura two-parameter model [26]. Bootstrap values were determined by 1000 replicates.

### Statistical analysis

Measurement data are represented as the mean ± SD, and analyzed using the unpaired Student’s *t*-test. Difference between rates was evaluated by Chi-square test and Fisher's exact test. *P* <0.05 were considered statistically significant. The cartogram was drawn using Excel software (Microsoft Co., USA). All statistical analyses were performed using the SPSS 13.0 software (SPSS Inc., USA).

## Results

### Virological surveillance of HCoV and 7 common respiratory viruses

Specimens from a total of 13048 patients were collected and analyzed over a 5-year-period from July 2010 to June 2015 in Guangzhou, south China, for 8 respiratory viruses, namely, HCoV, Influenza, PIV, RSV, HMPV, HRV, AdV and HBoV. The surveillance results showed that 5127 (39.29%) were found positive for at least one virus and 4727 (36.22%) were infected by more than one virus. As shown in Table 1, among the 13048 patient with fever and respiratory infection symptoms, 14.97% of patients were positive for Flu, 4.60% for PIV, 10.92% for RSV, 2.41% for hMPV, 5.99% for HRV, 5.39% for ADV and 1.45% for HBoV. HCoV was detected in 294 samples (2.25%, with median age 40 years) by real-time PCR, including 251 inpatients (detection rate 2.95%) and 43 outpatients (detection rate 0.95%).

**Fig 1:**
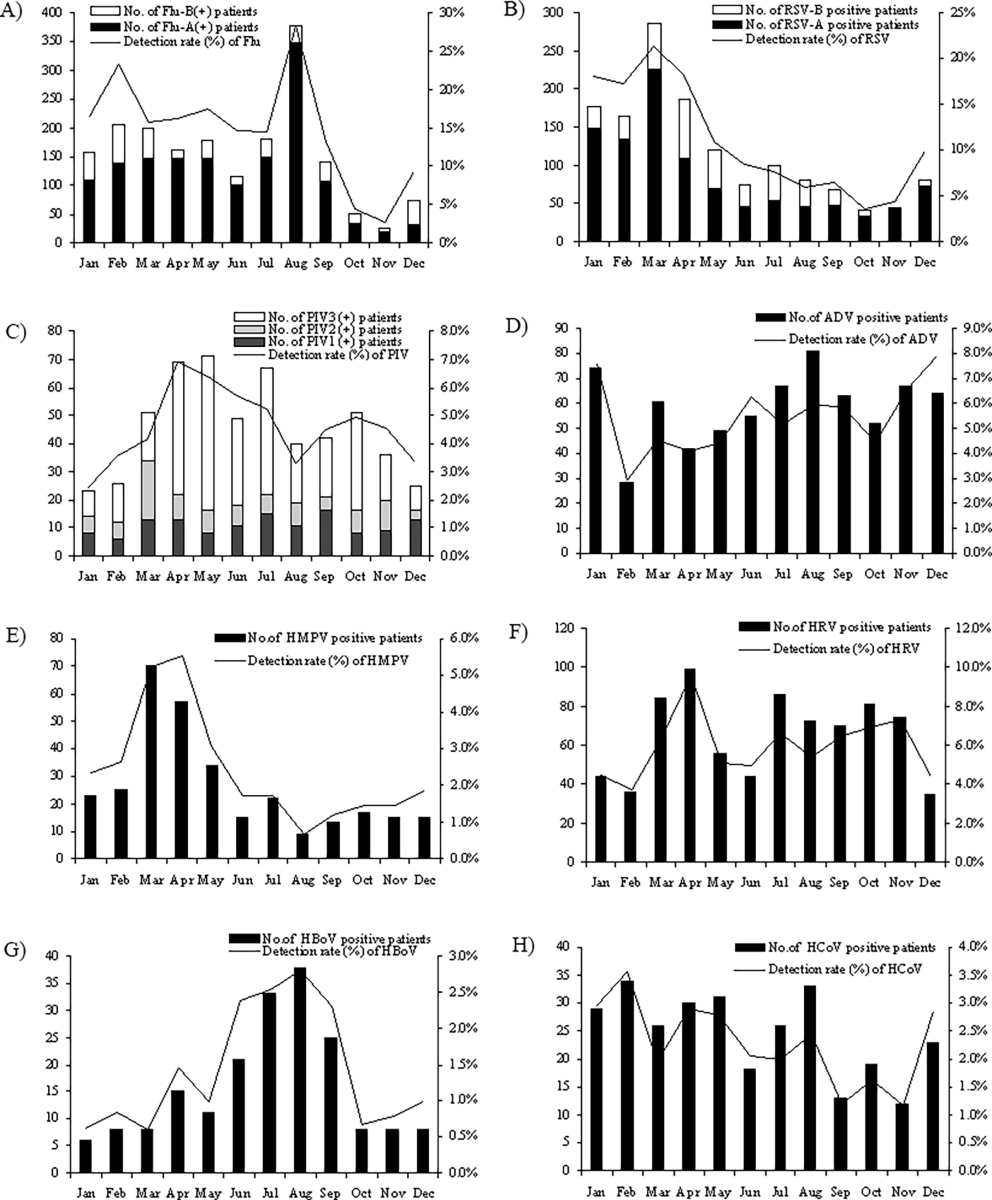
Monthly distribution of human coronavirus (HCoV) and other 7 common respiratory viruses from 13048 patients with acute respiratory infection symptoms in Guangzhou from July 2010 to June 2015. Virus-positive patient number of each month and the monthly detection rate (% of monthly detected cases) were shown. (A) influenza virus (Flu) type A and type B; (B) respiratory syncytial virus (RSV) type A and type B; (C) parainfluenza virus (PIV) type1-3; (D) adenovirus (ADV); (E) human metapneumovirus (HMPV); (F) human rhinovirus (HRV); (G) human bocavirus (HBoV); (H) human coronavirus (HCoV).

The monthly distributions of HCoV and 7 other common respiratory viruses tested in patients with acute respiratory infection symptoms from July, 2010 to June, 2015 were shown in Fig 1. Influenza virus was the most commonly detected respiratory virus which showed its peak of detection rate in August and another lower peak in February. Similarly, the prevalent peak of HBoV was in summer, with its highest detection rate appeared in July and August. RSV was mainly prevalent in spring and winter with its peak appeared in January to March. PIV was mainly prevalent in spring and autumn, and the detection rate was relatively low in winter. HMPV also was mostly detected in spring, with its peak in March and April. HRV and ADV were prevalent throughout the year, with their highest detection rate in April and December, respectively (see Fig 1). HCoV was also prevalent throughout the year, and highest detection rate appeared in February (3.56%, Fig 1).

**Fig 2:**
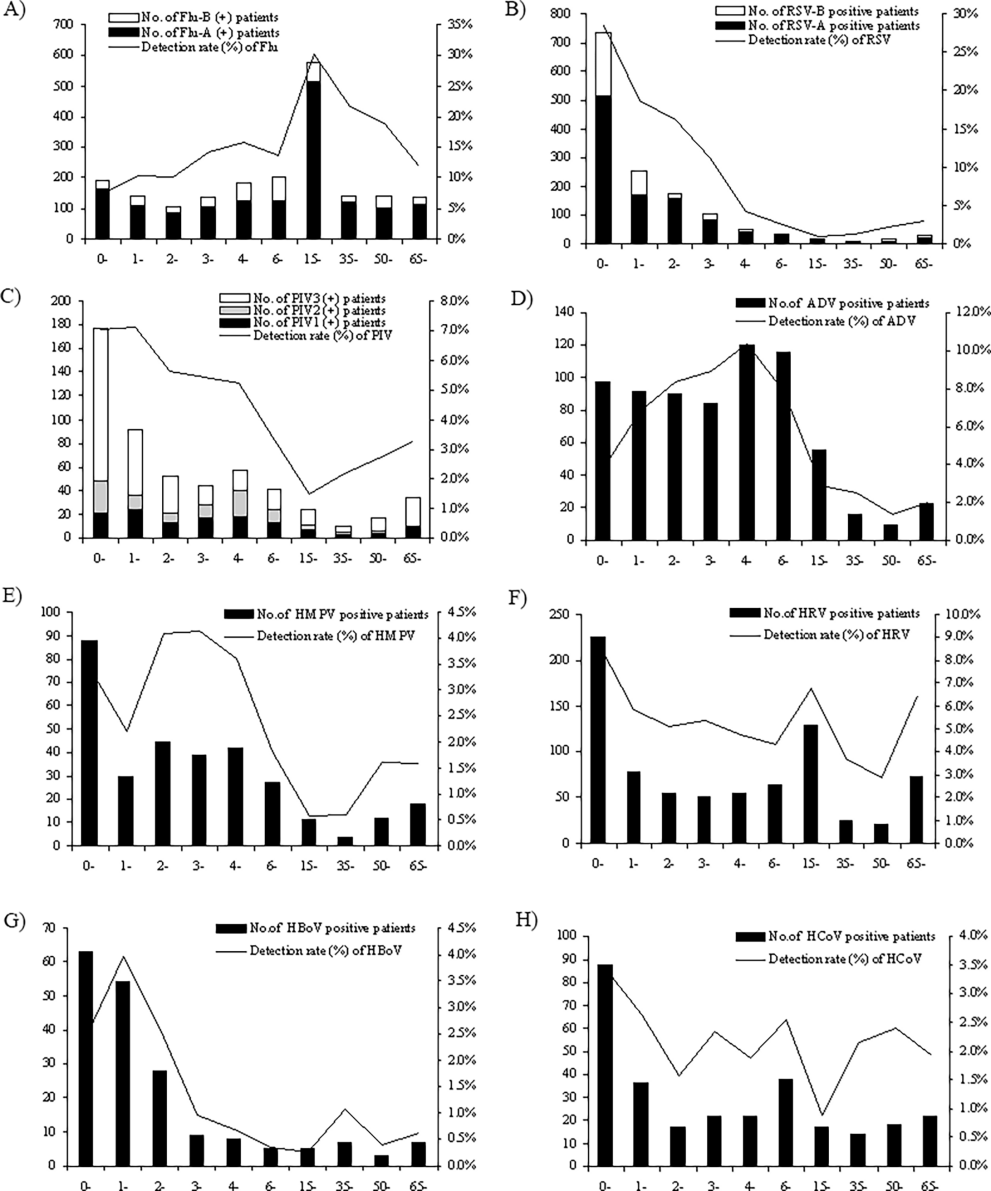
Age distribution of human coronavirus (HCoV) and other 7 common respiratory viruses from 13048 patients with acute respiratory infection symptoms in Guangzhou from July 2010 to June 2015. The number of virus-positive patients in different age groups, and the corresponding detection rate (% of detected cases in corresponding age group) were shown. (A) influenza virus (Flu) type A and type B; (B) respiratory syncytial virus (RSV) type A and type B; (C) parainfluenza virus (PIV) type1- 3; (D) adenovirus (ADV); (E) human metapneumovirus (HMPV); (F) human rhinovirus (HRV); (G) human bocavirus (HBoV); (H) human coronavirus (HCoV).

Patients enrolled in this study aged from 1 day to 103 years, including 8602 children and 4446 adult patients with a median age of 50 years. The total infection rate of common respiratory virus in children is 46.59% (4008/8602), as compared to that of 32.91% (1463/4446) in adults. The age distributions of 8 common respiratory viruses were shown in Fig 2. For most of the screened respiratory viruses, the infection rate of pediatric patients was higher than adult patients (*P*<0.05) except influenza virus, which tended to infect adults and mostly detected in age group 15–35 years (Table 1 and Fig 2). In contrast to influenza virus, RSV, PIV and HBoV tended to mostly infect infants and toddlers younger than 3 years of age with a few adult infection. ADV and hMPV tends to infect young children and mostly detected in 3–6 years old group (Table 1 and Fig 2). Similarly, HCoV mainly infected children under 15 years old (2.81% in 0–3 years infants and toddlers and 2.71% in 7–15 years elder children), but the detection rate was relatively lower in 3–6 younger children group (2.10%).

### Clinical characteristics of HCoV positive cases and epidemiological distribution of HCoV species

Among the 13048 cases, 2.25% (294 cases) were detected as HCoV positive, including 192 males (2.41%) and 102 females (2.01%). No significant difference existed between the detection rates of male and female (*P*>0.05). Among the 294 HCoV positive patients, 251 were inpatients (85.4% of the HCoV positive patients) and 43 were outpatients (14.6% of the HCoV positive patients). Significant difference existed between the detection rate of inpatient and outpatient (*P*<0.01). The odds of infection with HCoV resulting in severe disease (or admission) were 3.17 (95% CI 2.29–4.39). The common symptoms of patients detected as HCoV positive included cough (83.33%), fever (65.31%), sputum (30.61%), rhinorrhea (30.27%), tachypnea (12.24%) and sore throat (9.86%). Other symptoms included diarrhea (3.74%), dyspnea (2.04%), and chest pain (1.36%). Most of the HCoV positive patients were young children not in nursery (156 out of 294 HCoV positive cases, 53.06%). 39 were young children in school nursery (13.27% of the HCoV positive patients) and 26 were school students (8.84% of the HCoV positive patients).

Four species of HCoVs were detected in patients with acute respiratory infection symptoms during 2010–2015 in Guangzhou, south China, namely, 229E, OC43, NL63 and HKU1. Of the 13048 samples collected during 2010–2015 in Guangzhou, 177 were detected as OC43 positive (detection rate 1.36%), 49 as 229E positive (0.38%), 44 as NL63 positive (0.34%), and 23 as HKU1 positive (0.18%). There was 1 case (22 years old female) detected as coinfected by both HKU1 and OC43, but there was no evidence that co-infection resulted in more severe symptoms. Of the 294 total HCoV positive cases, 60.20% were OC43, 16.67% were 229E, 14.97% were NL63 and 7.82% were HKU1. The most prevalent HCoV in Guangzhou from 2010 to 2015 was OC43.

**Fig 3:**
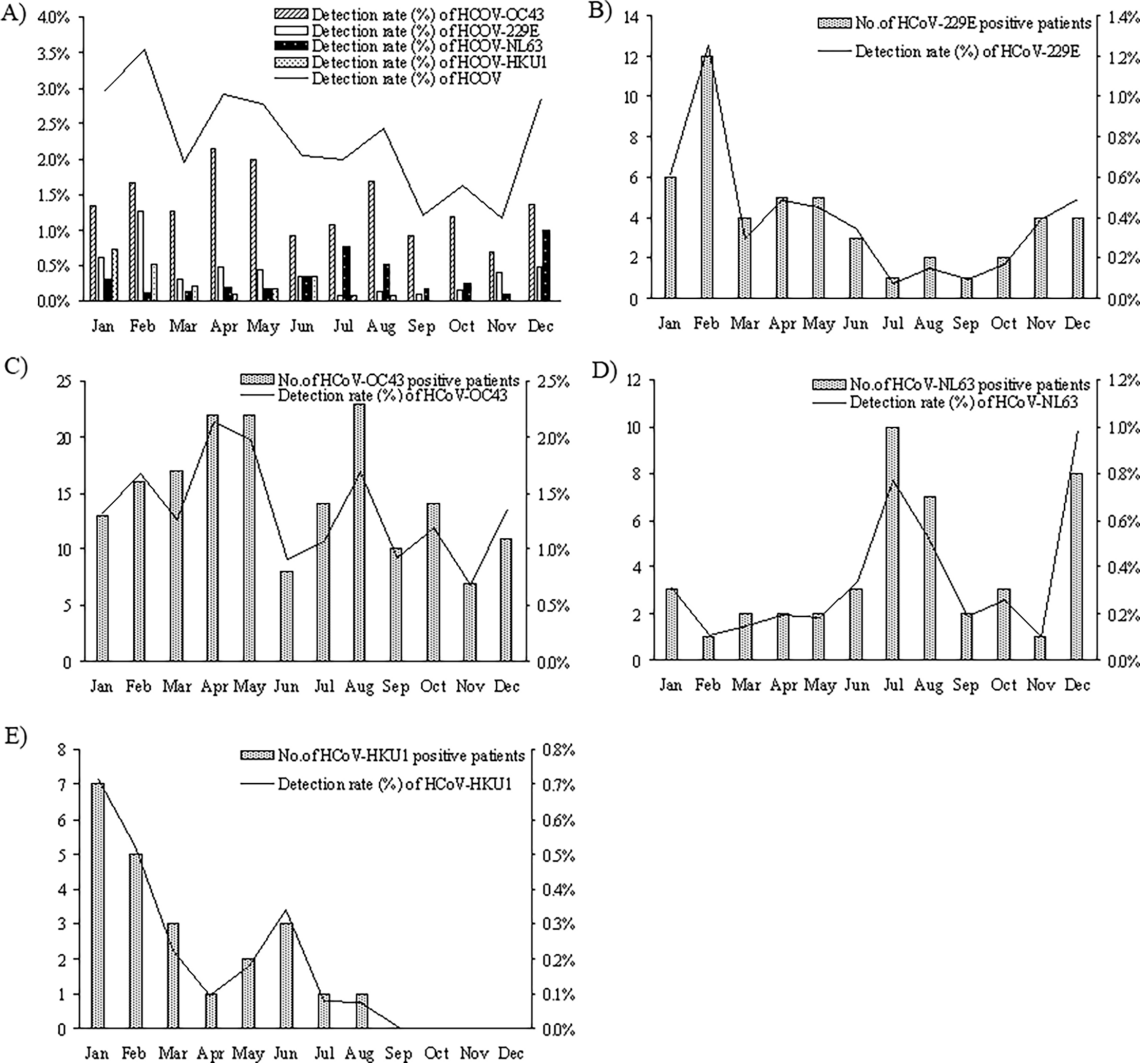
Monthly distributions of HCoV and its species from 13048 patients with acute respiratory infection symptoms in Guangzhou during 2010–2015. Four HCoV species (229E, OC43, NL63 and HKU1) were detected in Guangzhou during 2010–2015. The number of positive patients and the monthly detection rate (% of monthly detected cases) of total human coronavirus (HCoV) and the four detected HCoV species were shown. (A) the monthly detection rate (%) of total human coronavirus (HCoV) and the four detected HCoV species; (B)-(E) HCoV-229E, HCoV-OC43, HCoV-NL63 and HCoV-HKU1 positive case number of each month and the monthly detection rate.

The month distribution of total HCoV and the 4 detected species was shown in Fig 3 and S1 Fig. From the month distribution of total HCoV, we can see that HCoV was mainly epidemic in winter and spring, but differences existed in different species. HCoV-OC43 can be detected throughout the year, and its detection rate was relatively higher in spring (April and May), but lower in winter, but no significant difference existed in detection rates of different months (χ^2^ = 17.089, *P>*0.05). The epidemic peak of 229E appeared in February whereas the detection rate was much lower for the rest of the months (χ^2^ = 30.932, *P*<0.05). NL63 was mainly detected in summer (July to August) and winter (December), and differences existed in detection rates of different months (χ^2^ = 25.872, *P*<0.05). The peak of HKU1 appeared in January to February, but no positive cases were detected in September to December, significant differences also existed in detection rates of different months (χ^2^ = 33.376, *P*<0.05, see Fig 3 and S1 Fig).

**Fig 4:**
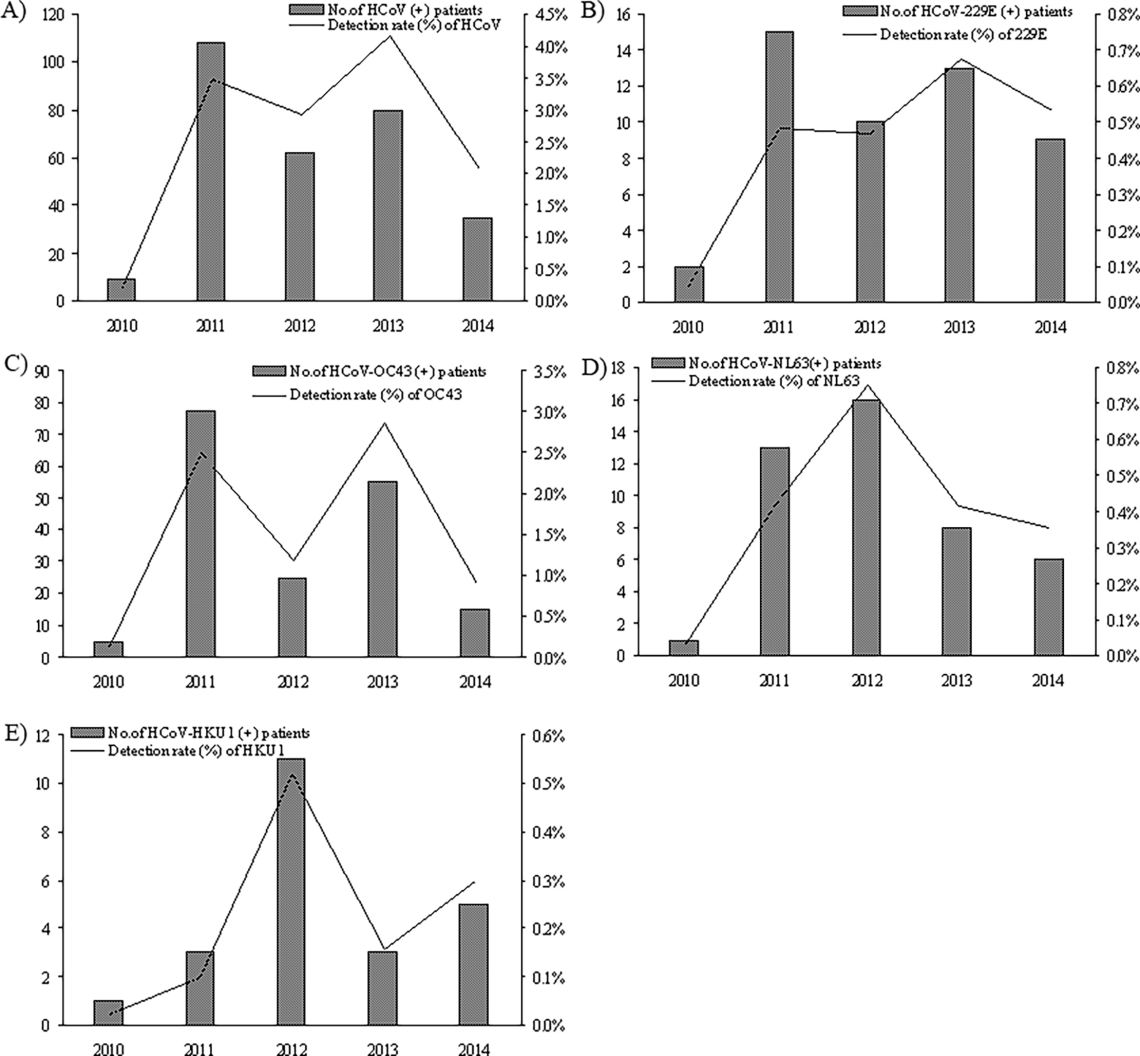
Year distributions of HCoV and its species from 13048 patients with acute respiratory infection symptoms in Guangzhou from July 2010 to June 2015. The number of positive patients and the detection rate (% of detected cases in the corresponding year) were shown. The time span of the year on x-axis referred to a 12 months span from July of corresponding year to June of the next year. (A)-(E) total HCoV, HCoV-229E, HCoV-OC43, HCoV-NL63 and HCoV-HKU1 positive case number and the detection rate of each year during 2010–2015.

The 5 year distribution of total HCoV and the detected species from 2010 to 2015 in Guangzhou was shown in Fig 4 and S1 Fig, and the sample numbers and detection rate of total HCoV and detected species in each year was shown in Table 4 For HCoV and all the species, 2010 was a low infection year, but the infection rate dramatically increased in 2011 and from then on, kept a relatively higher prevalence. From the 5 year distribution, we can see that total HCoV infection rate was relatively low in 2010, 2012 and 2014, and high in 2011 and 2013 (Fig 4A), showing a peak-valley distribution trend (χ^2^ = 136.418, *P*<0.05). The same trend can be observed for HCoV-OC43 (χ^2^ = 112.955, *P*<0.05) and HCoV-229E (χ^2^ = 19.255, *P*<0.05), but it is interesting to see that for HCoV-NL63 and HCoV-HKU1, a higher epidemic peak can be seen in 2012 and 2014 (for HCoV-HKU1) (Fig 4, χ^2^ = 24.125 and 22.110 respectively, *P*<0.05), when detection rates of HCoV-OC43 and HCoV-229E were relatively lower.

**Table 4:** Sample numbers and detection rate of total HCoV and detected species in each year during 2010–2015.

Year*	Collected samples	Positive samples and detection rate (%)				
		HCoV	229E	OC43	NL63	HKU1
2010	4196	9(0.21)	2(0.05)	5(0.12)	1(0.02)	1(0.02)
2011	3108	108(3.47)	15(0.48)	77(2.48)	13(0.42)	3(0.10)
2012	2128	62(2.91)	10(0.47)	25(1.17)	16(0.75)	11(0.52)
2013	1924	80(4.16)	13(0.68)	55(2.86)	8(0.42)	3(0.16)
2014	1692	35(2.07)	9(0.53)	15(0.89)	6(0.35)	5(0.30)
Total	13048	294(2.25)	49(0.38)	177(1.36)	44(0.34)	23(0.18)

*: Samples were collected from patients with acute respiratory infection symptoms in Guangzhou from July 2010 to June 2015. The year span in the table was from July of the referred year to June of the next year.

**Fig 5:**
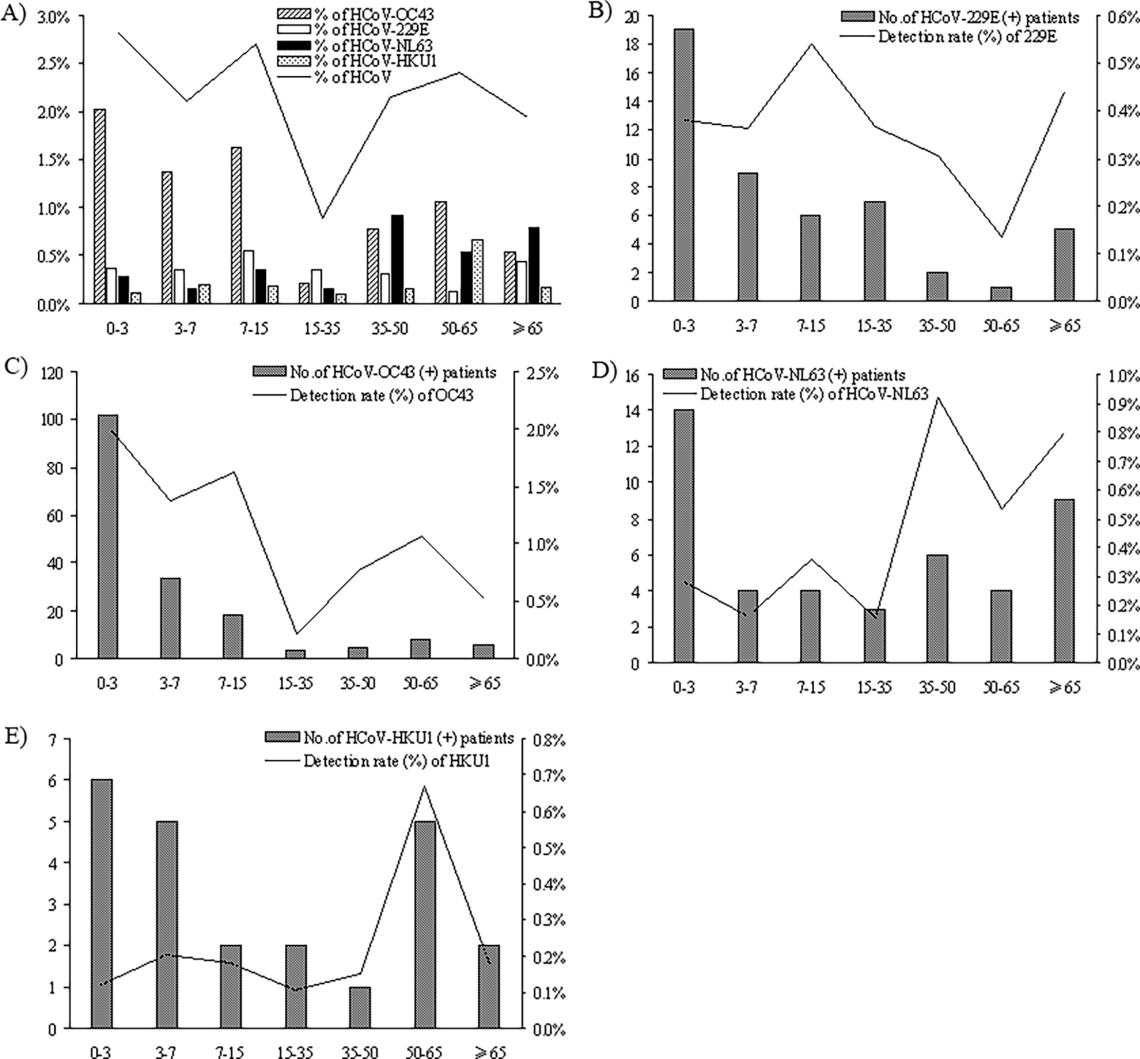
Age distribution of HCoV and its species from 13048 patients with acute respiratory infection symptoms in Guangzhou during 2010–2015. The number of positive patients in different age groups and the corresponding detection rate (% of detected cases in corresponding age group) were shown. (A) the detection rate (%) of total human coronavirus (HCoV) and the four detected HCoV species in different age groups; (B)-(E) HCoV-229E, HCoV-OC43, HCoV-NL63 and HCoV-HKU1 positive case number and detection rate in different age groups.

The age distribution of total HCoV and the 4 detected species was shown in Fig 5. For total HCoV, children (<15 years) and old people (>50 years) were both high risk groups, but for specific HCoVs, differences existed. From the detection rate, we can see that HCoV-OC43 mainly infected <3 years infants and toddlers. HCoV-229E more likely infected elder children of 7–15 years. As a contrast, HCoV-NL63 more likely infected adults of 35–50 years, and HCoV-HKU1 tended to infect old people of 50–65 years. Aged people of >65 years were also high risk group to infect HCoV-229E and HCoV-NL63.

### Co-infection

Of the 294 HCoV positive patients, 101 patients (34.69% of the HCoV positive patients) were co-infected by at least one other common respiratory virus. Among them, 91 cases (91/101, 90.20% of the co-infected patients) were double infection, and 10 cases (10/101, 9.80%) were triple infection (Table 5). Influenza virus was the most common co-infecting virus (30/101, 29.70%), and the next common co-infecting virus was RSV (23/101, 22.77%), as shown in Table 5. Of the 101 co-infection cases, 10 cases were emergency/outpatients, and 91 were inpatients. The co-infection rate was 23.26% (10/43 HCoV-positive outpatients) for emergency/outpatient, and 36.25% (91/251 HCoV-positive inpatients) for inpatient. There was no significant difference in the co-infection rates between emergency/ outpatients and inpatients (*P*>0.05), and between male and female (*P*>0.05). No correlation was found between co-infection and clinical symptoms, and among the 101 HCoV co-infection cases, 58 was diagnosed as lower respiratory tract infection, not statistically higher than that of HCoV single positive patients (58/101 vs. 93/193, *P*>0.05).

**Table 5:** Co-infection cases of HCoV and 7 other common respiratory viruses.

Co-detected viruses	Patient No. (% of total co-detected cases)
HCoV+Flu	30 (29.70%)
HCoV+RSV	23 (22.77%)
HCoV+PIV	12 (11.88%)
HCoV+HRV	10 (9.90%)
HCoV+HMPV	7 (6.93%)
HCoV+ADV	6 (5.94%)
HCoV+HBoV	3 (2.97%)
HCoV+RSV+Flu	2 (1.98%)
HCoV+Flu+PIV	1 (0.99%)
HCoV+Flu+ADV	1 (0.99%)
HCoV+RSV+PIV	1 (0.99%)
HCoV+RSV+HRV	2 (1.98%)
HCoV+PIV+ADV	1 (0.99%)
HCoV+HMPV+HRV	2 (1.98%)
Total	101 (100%)

Human coronavirus (HCoV) and other 7 common respiratory viruses including influenza (Flu), respiratory syncytial virus (RSV), parainfluenza (PIV), adenovirus (AdV), human metapneumovirus (HMPV), human rhinovirus (HRV) and human bocavirus (HBoV) were screened during 2010–2015 in Guangzhou, China. Cases number (%) of HCoV co-infected with other respiratory were shown. Of the 294 HCoV positive cases, 101 cases were co-detected by other common respiratory viruses, with 91 double infection cases and 10 triple infection cases.

### Sequences and phylogenetic analysis

**Fig 6:**
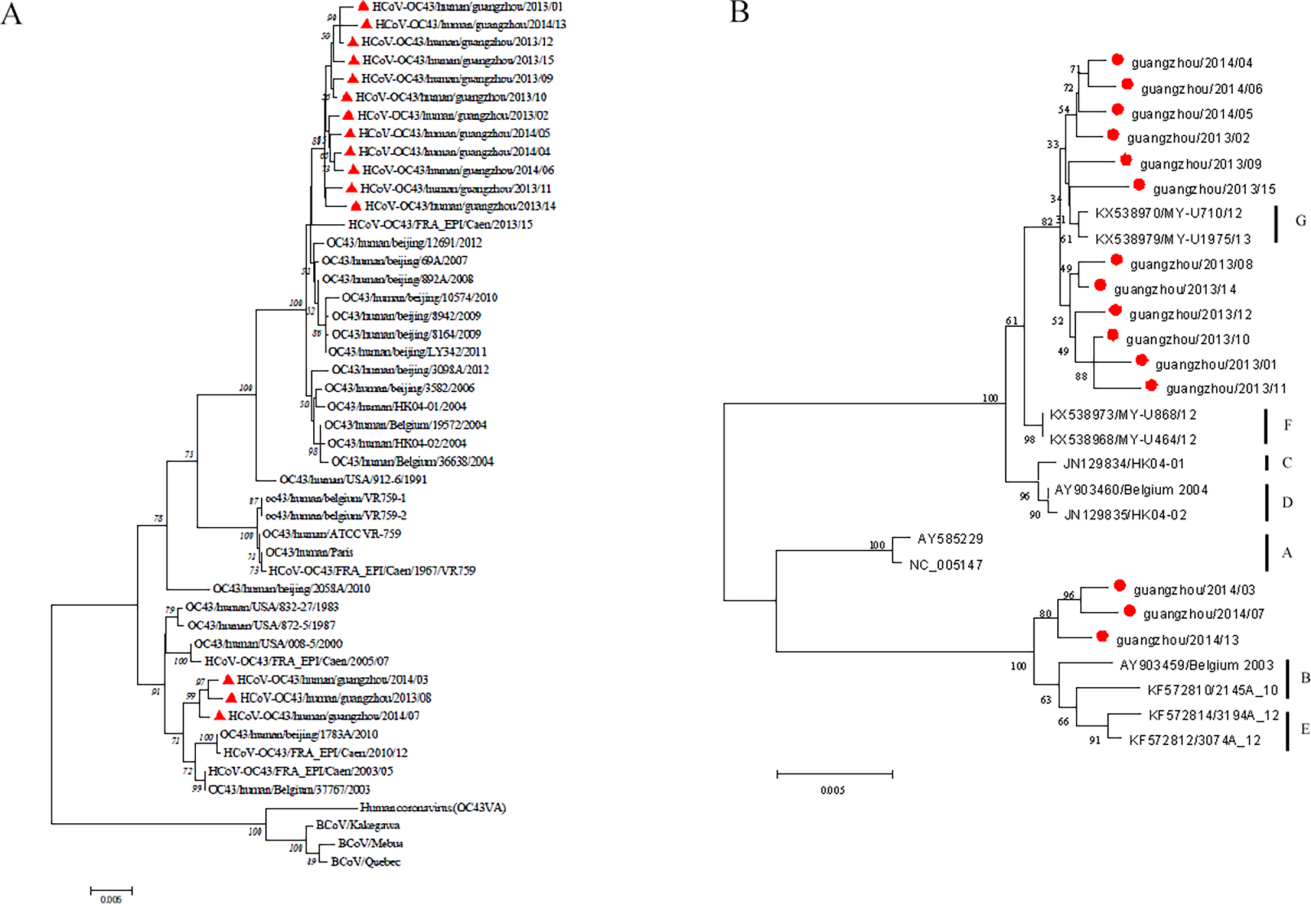
Phylogenetic analysis of 15 HCoV-OC43 strains detected in Guangzhou from 2013–2014 based on partial S gene sequence. Phylogenetic tree with 1,000 bootstrap replicates was generated using the neighbor-joining method with Mega 5.0 software. Trees were constructed using the maximum-likelihood method based on sequences of partial S genes of 2524nt. (A) Phylogenetic analysis of 15 HCoV-OC43 strains detected in Guangzhou from 2013–2014. Partial S gene sequences of 15 strains (labeled with red triangle) were comparatively analyzed with reference sequences of 33 representative HCoV-OC43 strains in the GenBank database. The 15 strains could be divided into 2 clusters. (B) Genotype analysis of the 15 strains identified in this study (presented with red circle). A, B, C, D, E, F and G represent known genotypes of OC43. Of the 15 OC43 Guangzhou strains from 2013–2014, 3 strains were closer to genotype B, whereas the other 12 strains were closer to the new genotype G.

To understand the variation of HCoV during 2010–2015 in Guangzhou, 15 HCoV-OC43 positive samples of 2013–2014 were selected for RT-PCR amplification and sequencing of S gene. Totally 2524 nt of OC43 S gene were successfully amplified and sequenced. Bovine CoV (accession no. U00735) was used as outgroup sequence, which was not displayed in the figure. Phylogenetic analysis showed that the 15 strains could be divided into 2 clusters, and 12 strains of which were most related to the strain from France (GI: 721684923), and the remaining 3 strains were most related to the strains from Beijing (GI: 744516692) and France (GI: 721684917), as shown in Fig 6A. Because in the phylogenetic tree of S gene, these 12 strains formed a separate cluster, distant from the other 3 strains, we further analyzed the genotype of these 12 strains. Eighteen other S gene sequences of OC43 were used as reference strains, including ATCC-VR759 (AY585229, AY585228 and NC005147) as genotype A reference, BE-03 (AY903459) as genotype B reference, HK04-01 (JN129834) as genotype C reference, HK04-02 (JN129835) and OC43 BE-04 (AY903460) as genotype D reference, KF572812 as genotype E reference, and Malaysia strains KX538973 (MY-U868/12) and KX538970 (MY-U710/12) as genotype F and G reference, respectively. It was shown that these distinct 12 strains were more close to the novel genotype G (Fig 6B), whereas the other 3 strains were close to genotype B. The partial S gene sequences of 15 strains of 2013–2014 in Guangzhou were deposited in GenBank under accession numbers KX447776- KX447790.

## Discussion

Prior to the SARS-CoV outbreak, coronaviruses were thought to cause mild, self-limiting respiratory infections in humans [4,8]. But the emergence of SARS-CoV and MERS-CoV changed the recognition. The high pathogenicity of SARS-CoV brought renewed interest and concerns in this virus family, and further research on the origin of SARS- and MERS-CoV revealed the possibility of coronavirus variation and transmission from animal hosts to human beings [1–2,11,27–28]. Therefore, the surveillance of coronavirus in humans as well as in animals is very necessary and important for HCoV variation research and infection control. However, to the best of our knowledge, there is very limited report of HCoVs molecular epidemiology in Guangzhou and the variation report about HCoV is few. Therefore, in this study, the molecular epidemiological characteristics of HCoVs in pediatric and adult patients with acute respiratory infection symptoms in Guangzhou from 2010–2015 were investigated, and the phylogenetic and genotypic analysis of S gene of the most prevalent HCoV species OC43 was performed, and the epidemic of the novel OC43 genotype G in Guangzhou was for the first time observed.

We collected totally 13048 throat and nasal swabs from patients with acute respiratory infection symptoms during 2010–2015 in Guangzhou, and HCoV and its species were detected with other 7 common respiratory viruses. Totally 39.29% of the patients were detected as positive for at least one of the 8 respiratory viruses. The detection rates, age and month distributions of Influenza, PIV, RSV, HMPV, HRV, AdV and HBoV were consistent with our previous studies and other reports (Figs 1 and 2) [14,25,29]. HCoV was detected in 2.25% (294 positive) patients with respiratory infection symptoms, with the detection rate as 2.95% of inpatients and 0.95% of outpatients. The detection rate was significantly higher in inpatient than outpatient (*P*<0.01), including adult and children (*P*<0.05), and the detection rate was especially higher in infant inpatients (Table 1), and the odds of HCoV infection resulting in admission or severe disease were 3.17, showing that HCoV infection is dangerous especially for infants and toddlers. Real-time RT-PCR was used for HCoVs detection, to increase the sensitivity and avoid cross contamination and false positive. The higher sensitivity of real-time PCR method may contribute to the higher detection rate of HCoV in our study compared with Jinan and Hongkong that used traditional RT-PCR for HCoV screening [30–32], and may be a better method in HCoV surveillance. Nevertheless, because HCoV detection rate varies in different regions and countries [30–35], another reason for different detection rates may also lie in region distribution. Totally 4 HCoV species including HCoV-229E, OC43, NL63 and HKU1 were detected during 2010–2015 in Guangzhou, and no SARS and MERS-CoV was detected, confirming that the outbreak of highly pathogenic MERS-CoV in year 2015 in South Korea did not spread to Guangzhou. Of the locally epidemic HCoVs, OC43 was the most commonly detected, followed by 229E and NL63, and HKU1 detection rate was the lowest.

From the monthly distribution and year distribution, we found that HCoV-OC43 is the main prevalent HCoV in Guangzhou during 2010–2015. Different HCoVs showed different epidemic months and seasons (Fig 3 and S1 Fig). OC43 was prevalent throughout the year, whereas 229E was prevalent mainly in winter (especially in February). NL63 was most epidemic in summer and winter, whereas the peak of HKU1 appeared in winter (January and February) and disappeared in September to December. The 5 year distribution of HCoVs during 2010–2015 in Guangzhou shows an every other year trend of epidemiology, a peak-valley distribution, that is, relatively lower in 2010, 2012 and 2014, and higher in 2011 and 2013 (Fig 4). Similar phenomena could be found in the study of Dare RK et al [36], but there was only 2 years of data in that study [36]. Therefore, data from continuous of surveillance is very important to reveal the pattern of HCoV epidemiology. Further analysis found that this trend mainly came from OC43 year distribution, and secondly, 229E distribution. However, in 2012 and 2014, when the detection rates of OC43 and 229E were relatively low, the infection rates of NL63 and HKU1 were high, and both of them showed a peak in 2012 (Fig 4). From the 5 year distribution data, we can see that in Guangzhou the infection rate of HCoV was dramatically increased after 2010, and take on a peak-valley pattern (Fig 4 and Table 4). More years of surveillance are needed to confirm this HCoV epidemic pattern in Guangzhou.

Similar to RSV, PIV, HMPV and HBoV, HCoVs tend to infect children (Fig 2) [14, 25, 29, 31–34]. Our results showed that young children not in nursery had the highest risk of HCoV infection (53.06% of the HCoV positive patients). The reason may be that most children <3 years old in China are not in nursery, and they usually stay at home with their family guardians. Since HCoV infection is common in adults and elder people, we deduce that the most of sporadic infection should come from the guardians of those young children, and therefore protective measures for children guardians are very important for HCoV prevention and control. From the age distribution, we found that <15 year old children and >50 year elder people were both high risk groups of HCoV infection, and the risk was particularly higher in infants under 1 year old (Fig 2), but there were differences between HCoVs. OC43 mainly infected <3 years infants and toddlers, whereas 229E more likely infected elder children of 7–15 years. NL63 more likely infected middle aged adults of 35–50 years, but HKU1 tended to infect elder people of 50–65 years. These epidemiological characteristics may help to understand the pathogenicity of HCoV and prevent HCoV infection.

It is well known that HCoV is one of the most likely co-infected viruses [31–34], therefore in our study, 7 other common respiratory viruses were detected simultaneously. It was found that 34.69% of the HCoV positive patients were co-infected by at least one of other respiratory viruses (Table 5). Most of the co-infections (91/101) were double infection and 10 cases (10/101, 9.8%) were triple infection. Influenza and RSV were the most common respiratory viruses that co-infected with HCoV. Parainfluenza virus and rhinovirus were also common co-infected viruses. Although co-infection rate was high for HCoV, there is no obvious evidence that co-infection could increase the risk of patient hospitalization, or the chance of lower respiratory tract infection (*P*>0.05), and no correlation was found between co-infection and clinical symptoms.

HCoV-OC43 belongs toβ-genera of coronavirus, the same genera also includes high pathogenic SARS-CoV and MERS-CoV [1–2,11–12,37]. In this study, HCoV-OC43 was selected for variation analysis for the reason that it was the most prevalent HCoV in Guangzhou during 2010–2015, and it was most variable species among the 4 detected HCoVs [38–40]. In this study, 15 strains of OC43 from 2013–2014 were chosen for phylogenetic analysis of based on partial S gene sequences. It was shown that the 15 OC43 strains could be divided into 2 clusters, and 12 strains of which were most related to the strain from France (GI: 721684923), and the remaining 3 strains to Beijing (GI: 744516692) and France (GI: 721684917). Because these 12 strains formed a separate cluster, which was distant from the other 3 strains (Fig 6A), we further analyzed the genotype of these 12 strains.

Traditionally, 4 genotypes (A, B, C and D) have been identified based on the viral genome and the phylogeny of the main structural genes, S, RNA-dependent RNA polymerase (RdRp), and nucleocapsid (N) genes [38]. In 2015, a new genotype E was identified which was reported to have arisen due to natural recombination [39]. Recently, 2 new genotypes of OC43 were reported as F and G genotypes in Malaysia [40], indicating that OC43 were evolving continuously. However, due to the limited availability of HCoV-OC43 sequences, the variation of HCoV-OC43, especially its genotyping, remained to be further elucidated. Therefore, in this study, we analyzed the genotypes based on partial S gene sequences of 15 HCoV-OC43 strains from positive samples during 2013–2014 using PCR amplification and sequencing. We found that 3 strains were closer to genotype B, but the remaining 12 strains were more close to the newly defined genotype G in the phylogenetic trees (Fig 6B). Recombinant analysis was also performed with negative results, indicating that genotype drift may be one of an important way for HCoV-OC43 to maintain its epidemic. This is for the first time that genotype G is reported to be epidemic as a dominant genotype during 2013–2014 in Guangzhou, south China. The epidemic of genotype G in Guangzhou may be a result of personnel exchange between China and Southeast Asia countries including Malaysia. Further complete genome sequencing will be needed to understand the phylogenic characteristics of these G genotype strains circulating in Guangzhou.

## Conclusion

In summary, we collected totally 13048 throat/nasal swab specimens from adults and children with fever and acute upper respiratory infection symptoms in Gunazhou, south China between July 2010 and June 2015, and the epidemiological features of HCoV were studied, and the phylogenetic features of HCoV-OC43 were analyzed. It was found for the first time that genotype B and genotype G was co-epidemic and the newly defined OC43 genotype G was a dominant genotype in Guangzhou during 2013–2014. Our findings may have significance for the prevention and control of HCoV infection, and provide insights into HCoV-OC43 variation and evolution.

## Supporting information

S1 Fig Distribution of HCoV-OC43, 229E, NL63 and HKU1 in each month and each year from July 2010 to June 2015 in Guangzhou. Totally 13048 throat swabs from patients with acute respiratory infection symptoms were screened for HCoVs by real-time RT-PCR. Four HCoVs as OC43, 229E, NL63 and HKU1 were detected. The number of positive patients and the monthly detection rate (% of monthly detected cases) of these four HCoV species were shown. (A) The positive numbers of four detected HCoV species; (B) The monthly detection rates of HCoV-OC43, 229E, NL63 and HKU1.
(TIF)

S1 Table The Primers and probes used for Flu, RSV, PIV, ADV, hMPV, HRV and HBoV Screening. (DOC)
