# Supplementary figures and images for: Epidemiology characteristics of human coronaviruses in patients with respiratory infection symptoms and phylogenetic analysis of HCoV-OC43 during 2010-2015 in Guangzhou
Source: PLoS One. 2018 Jan 29;13(1):e0191789. doi: 10.1371/journal.pone.0191789 (PMC5788356; doi:10.1371/journal.pone.0191789)

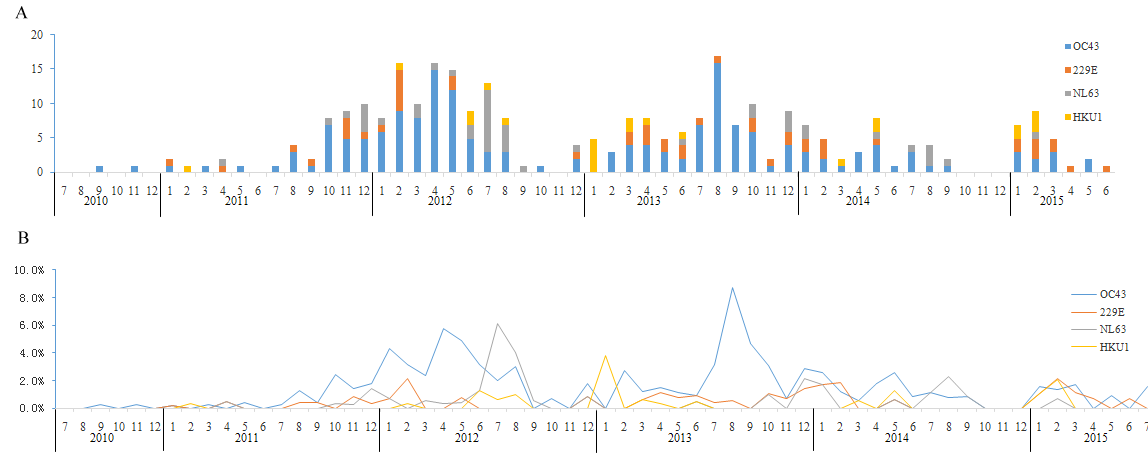

Supplement: S1 Fig — Totally 13048 throat swabs from patients with acute respiratory infection symptoms were screened for HCoVs by real-time RT-PCR. Four HCoVs as OC43, 229E, NL63 and HKU1 were detected. The number of positive patients and the monthly detection rate (% of monthly detected cases) of these four HCoV species were shown. (A) The positive numbers of four detected HCoV species; (B) The monthly detection rates of HCoV-OC43, 229E, NL63 and HKU1. (TIF) [file pone.0191789.s001.tif]
