# Supplementary material for: Epidemiology characteristics of human coronaviruses in patients with respiratory infection symptoms and phylogenetic analysis of HCoV-OC43 during 2010-2015 in Guangzhou
Source: PLoS One. 2018 Jan 29;13(1):e0191789. doi: 10.1371/journal.pone.0191789 (PMC5788356; doi:10.1371/journal.pone.0191789)
Supplement: S1 Table — (DOC) [file pone.0191789.s002.doc]

**S1 Table. The Primers and probes used for Flu, RSV, PIV, ADV, HMPV, HRV and HBoV** **Screening**

| Virus | Primer/probe | Sequence（5’-3’） | Target Gene | PCR product (bp) |
| --- | --- | --- | --- | --- |
| Flu-A | FluA-F | GACCRATCCTGTCACCTCTGAC | M2 | 106 |
|  | FluA-R | AGGGCATTYTGGACAAAKCGTCTA |  |  |
|  | FluA-Probe | FAM-TGCAGTCCTCGCTCACTGGGCACG-TAMRA |  |  |
| Flu-B | FluB-F | TGCCTACCTGCTTTMMYTRACA | M | 75 |
|  | FluB-R | CCRAACCAACARTGTAATTTTTCTG |  |  |
|  | FluB-Probe | FAM-TGCTTTGCCTTCTCCA-TAMRA |  |  |
| RSV-A | RSVA-F | GCTCTTAGCAAAGTCAAGTTGAATGA | N | 82 |
|  | RSVA-R | TGCTCCGTTGGATGGTGTATT |  |  |
|  | RSVA-Probe | FAM-ACACTCAACAAAGATCAACTTCTGTCATCCAGC-TAMRA |  |  |
| RSV-B | RSVB-F | GATGGCTCTTAGCAAAGTCAAGTTAA | N | 104 |
|  | RSVB-R | TGTCAATATTATCTCCTGTACTACGTTGAA |  |  |
|  | RSVB-Probe | FAM-TGATACATTAAATAAGGATCAGCTGCTGTCATCCA-TAMRA |  |  |
| PIV First round | PIV13-F | AGGWTGYSMRGATATAGGRAARTCAT | HA | PIV1：439  PIV2：297  PIV3：390  PIV4：174 |
|  | PIV13-R | CTWGTATATATATRTAGATCTKTTRCCTAGT |  |  |
|  | PIV2-F | TAATTCCTCTTAAAATTGACAGTATCGA |  |  |
|  | PIV4-F | ATCCAGARRGACGTCACATCAACTCAT |  |  |
|  | PIV24-R | TRAGRCCMCCATAYAMRGGAAATA |  |  |
| PIV Nested PCR | PIV13-F | ACGACAAYAGGAARTCATGYTCT |  |  |
|  | PIV1-R | GACAACAATCTTTGGCCTATCAGATA |  |  |
|  | PIV3-R | GAGTTGACCATCCTYCTRTCTGAAAAC |  |  |
|  | PIV24-F | CYMAYGGRTGYAYTMGAATWCCATCATT |  |  |
|  | PIV2-R | GCTAGATCAGTTGTGGCATAATCT |  |  |
|  | PIV4-R | TGACTATRCTCGACYTTRAAATAAGG |  |  |
| ADV | ADV-F | GCCSCARTGGKCWTACATGCAC ATC | Hexon | 301 |
|  | ADV-R | CAGCACSCCICGRATGTCAAA |  |  |
| HMPV | hMPV-F | CATGCCCACTATAAAAGGTCAG | L | 171 |
|  | hMPV-R | CACCCCAGTCTTTCTTGAAA |  |  |
| HRV First round | RV-F1 | CTCCGGCCCCTGAATRYGGCTAA | 5’NCR |  |
|  | RV-R1 | TCIGGIARYTTCCASYACCAICC |  |  |
| HRV Nested PCR | RV-F2 | ACCRASTACTTTGGGTRWCCGTG | VP4/ VP2 | 110 |
|  | RV-R2 | CTGTGTTGAWACYTGAGCICCCA |  |  |
| HBoV | HBoV-F | AGAGGCTCGGGCTCATATCA | NP1 | 88 |
|  | HBoV-R | TCTTCATCACTTGGTCTGAGGTCT |  |  |
|  | HBoV-probe | FAM-AGGAACACCCAATCARCCACCTATCGT-TAMRA |  |  |

Flu: influenza virus; RSV: respiratory syncytial virus; PIV: parainfluenza virus; ADV: adenovirus; hMPV: human metapneumovirus; HRV: human rhinovirus; HBoV: human bocavirus.
